# Predicting hemorrhagic transformation after large vessel occlusion stroke in the era of mechanical thrombectomy

**DOI:** 10.1371/journal.pone.0256170

**Published:** 2021-08-16

**Authors:** Takanori Iwamoto, Takaya Kitano, Naoki Oyama, Yoshiki Yagita

**Affiliations:** 1 Department of Stroke Medicine, Kawasaki Medical School, Okayama, Japan; 2 Department of Neurology, Osaka University Graduate School of Medicine, Osaka Japan; 3 Department of Neurology, Toyonaka Municipal Hospital, Osaka, Japan; Henry Ford Health System, UNITED STATES

## Abstract

Serum biomarkers are associated with hemorrhagic transformation and brain edema after cerebral infarction. However, whether serum biomarkers predict hemorrhagic transformation in large vessel occlusion stroke even after mechanical thrombectomy, which has become widely used, remains uncertain. In this prospective study, we enrolled patients with large vessel occlusion stroke in the anterior circulation. We analyzed 91 patients with serum samples obtained on admission. The levels of matrix metalloproteinase-9 (MMP-9), amyloid precursor protein (APP) 770, endothelin-1, S100B, and claudin-5 were measured. We examined the association between serum biomarkers and hemorrhagic transformation within one week. Fifty-four patients underwent mechanical thrombectomy, and 17 patients developed relevant hemorrhagic transformation (rHT, defined as hemorrhagic changes ≥ hemorrhagic infarction type 2). Neither MMP-9 (no rHT: 46 ± 48 vs. rHT: 15 ± 4 ng/mL, P = 0.30), APP770 (80 ± 31 vs. 85 ± 8 ng/mL, P = 0.53), endothelin-1 (7.0 ± 25.7 vs. 2.0 ± 2.1 pg/mL, P = 0.42), S100B (13 ± 42 vs. 12 ± 15 pg/mL, P = 0.97), nor claudin-5 (1.7 ± 2.3 vs. 1.9 ± 1.5 ng/mL, P = 0.68) levels on admission were associated with subsequent rHT. When limited to patients who underwent mechanical thrombectomy, the level of claudin-5 was higher in patients with rHT than in those without (1.2 ± 1.0 vs. 2.1 ± 1.7 ng/mL, P = 0.0181). APP770 levels were marginally higher in patients with a midline shift ≥ 5 mm than in those without (79 ± 29 vs. 97 ± 41 ng/mL, P = 0.084). The predictive role of serum biomarkers has to be reexamined in the mechanical thrombectomy era because some previously reported serum biomarkers may not predict hemorrhagic transformation, whereas the level of APP770 may be useful for predicting brain edema.

## Introduction

Reperfusion therapy such as intravenous recombinant tissue plasminogen activator (rt-PA) and mechanical thrombectomy improves the outcome of patients with acute ischemic stroke [1–3]. However, it is also known that reperfusion therapies may worsen the hemorrhagic transformation [3]. In addition, reperfusion injury may augment brain edema [4, 5], although several studies on the association between brain edema and reperfusion show conflicting results [6, 7]. Once severe hemorrhagic transformation or edema has developed, the prognosis is often not favorable [8, 9]. Therefore, the ability to rapidly identify patients who are at higher risk of hemorrhagic transformation and brain edema is necessary.

Hemorrhagic transformation and edema after cerebral infarction mainly result from disruption of the blood brain barrier (BBB) and increased vascular permeability due to injury and/or remodeling of cerebral vessels [10]. Several studies have previously revealed that biomarkers using peripheral blood are useful for predicting hemorrhagic transformation and brain edema [11–16]. For example, elevated matrix metalloproteinase-9 (MMP-9) levels are associated with hemorrhagic transformation [13], and elevated levels of endothelin-1 predict severe cerebral edema in stroke patients treated with rt-PA. However, these findings were obtained before mechanical thrombectomy became widely used, and whether these biomarkers predict hemorrhagic transformation and brain edema in the era of mechanical thrombectomy remains unclear.

In this prospective study, we examined whether these serum biomarkers were associated with the development of hemorrhagic transformation or brain edema in patients with large-vessel occlusion stroke in the anterior circulation. Furthermore, we investigated the association between biomarkers and functional outcomes.

## Materials and methods

This study complied with the Declaration of Helsinki, and the retrospective study protocol was approved by the institutional ethics committee of Kawasaki Medical School.

### Subjects

This was a single-center, prospective, observational study. We enrolled patients with acute ischemic stroke with internal carotid artery (ICA) or middle cerebral artery (MCA) occlusion who were admitted to Kawasaki Medical School Hospital within 24 hours of onset since June 2016. Patients aged 20 years or older who provided written informed consent were registered, and there was no upper age limit or minimum Alberta Stroke Program Early CT Score (ASPECTS) [17]. The study aimed to enroll 100 patients, and recruitment was censored in February 2020.

### Data collection

Data, including age, sex, medical history, pre-stroke modified Rankin scale (mRS) score [18], stroke subtype [19], National Institutes of Health Stroke Scale (NIHSS) score, sites of occluded vessels, laboratory findings, and ASPECTS [17], which were determined based on computed tomography (CT) and magnetic resonance imaging (MRI) at admission, and the use of intravenous recombinant tissue plasminogen activator (rt-PA), were acquired from the patients’ medical records. If both CT and MRI were performed, MRI findings were prioritized to determine the ASPECTS value.

Reperfusion was evaluated at the end of the endovascular procedures and/or 24 ± 12 h after admission using magnetic resonance angiography (MRA). Successful angiographic reperfusion was identified based on grade 2b or greater using the expanded thrombolysis in cerebral infarction (eTICI) system in digital subtraction angiography [20], and grade 2 or greater using modified Mori grade in MRA [21].

Mechanical thrombectomy was defined as a procedure with arterial catheterization with a stent retriever or catheter aspiration, or both, with or without the delivery of a thrombolytic agent. The decision on whether to perform endovascular treatment and the type of treatment for each patient was left to the discretion of the attending physicians. The total number of device passes attempted before angiographic reperfusion or at the end of the procedure was reviewed for each patient. We classified the techniques as follows: “catheter aspiration,” “stent retriever,” and “combined”.

### Outcomes

The primary outcome of this study was hemorrhagic transformation within a week of admission. The secondary outcomes were the development of malignant edema, neurological deterioration, and functional outcomes at three months after stroke onset. CT or MRI scans were performed 72 ± 12 h after admission to evaluate hemorrhagic transformation and edema. In addition, evaluations were performed in accordance with clinical necessity. According to the European-Australasian Acute Stroke Study II definitions [22], hemorrhagic transformation was classified as hemorrhagic infarction type 1 (HI1) or type 2 (HI2), and parenchymal hematoma, as type 1, type 2, or remote parenchymal hematoma. As reported previously, relevant hemorrhagic transformation was defined as HI2 and any type of PH [13]. Malignant edema was defined as the presence of midline shift ≥ 5 mm [23, 24]. All brain images were evaluated by an experienced neurologist (T.K.) who was blinded to the levels of serum biomarkers and functional outcomes. Neurological deterioration was defined as an increase of ≥4 points in the NIHSS score within a week of admission. Functional outcome scores based on mRS were collected three months after stroke onset. The patient follow-up method has been described previously [25].

### Serum biomarker measurement

Serum samples were taken immediately upon admission at the emergency department before rt-PA administration or endovascular therapy and stored at < -80°C. Serum biomarker levels were determined using commercially available quantitative ELISA kits as follows: MMP-9, human ELISA kits for MMP-9 (PK-EL-64106, PromoCell, Heidelberg, Germany), amyloid precursor protein (APP) 770, Human ELISA kit of APP770 (#27736, Immuno-Biological Laboratories, Fujioka, Japan); endothelin-1, endothelin-1 ELISA kit (ADI-900-020A, EnzoLifescience, Lausen, Switzerland); S100B, Human ELISA kit of S100B (DY1820-05, DRG MedTek, Warsaw, Poland); and claudin-5, human claudin-5 ELISA kit (NBP2-75332, Novus Biologicals, CO, USA).

### Statistical analysis

Serum biomarker levels were compared according to hemorrhagic transformation type, presence or absence of midline shift, and neurologic deterioration. In addition, the association between serum biomarker levels and baseline characteristics such as age, NIHSS score, and ASPECTS was evaluated. Finally, we evaluated the association between serum biomarker levels and functional outcomes. Additionally, we analyzed the association between serum biomarkers and favorable outcome (mRS ≤ 2).

Continuous variables were reported as means and standard deviations or medians and interquartile ranges, while categorical variables were reported as numbers and percentages. Continuous variables were compared using t-tests. Correlations were tested using the Pearson correlation coefficient. Statistical significance was set at *P* <0.05. All analyses were performed using SAS on demand (SAS 9.4, SAS Institute Inc., Cary, NC, USA).

## Results

### Patients characteristics

**Fig 1:**
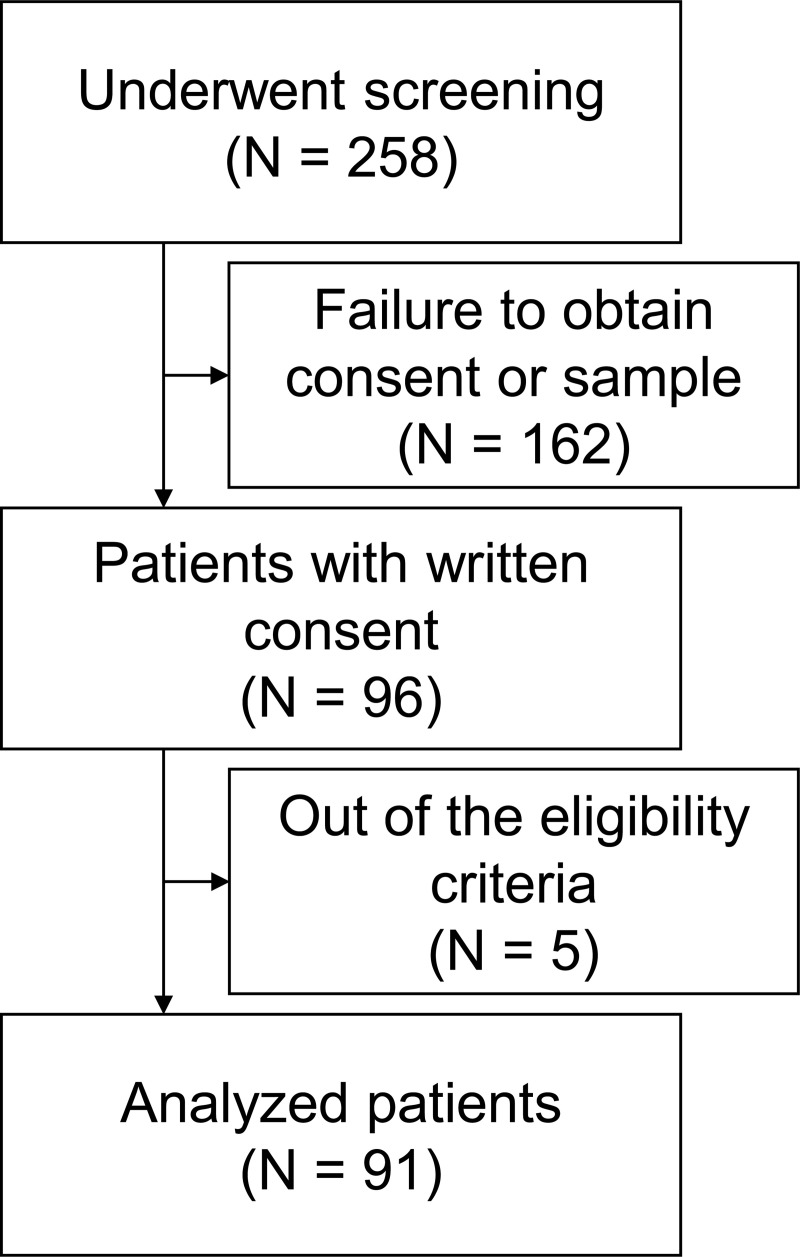
Patient selection. LVO, large vessel occlusion.

A total of 258 patients underwent screening. Among them, 96 patients with serum samples provided written informed consent. Four patients were excluded because they did not have ICA or MCA occlusion but other vessel occlusion, and one patient was excluded because of poor imaging quality. As a result, 91 patients were included in this study (Fig 1).

Baseline patient characteristics are shown in Table 1. The mean age was 77 years, and 60% of the patients were men. The mean NIHSS score was 18. The most frequently occluded vessel was the horizontal segment of the MCA (62%), followed by the intracranial ICA (27%) and the insular segment of the MCA (6%). Intravenous rt-PA was administered to 28 (31%) patients, and 54 (59%) patients underwent mechanical thrombectomy. As the first attempt, stent retriever was used in 18 (33%) patients, catheter aspiration in 20 (37%), and the combination of both in 16 (30%). Reperfusion was obtained within 36 h of admission in 67 patients (77%).

**Table 1:** Baseline patient characteristics.

Characteristics	n = 91
Age, years	77 ± 11
Male sex	55 (60%)
Hypertension	60 (66%)
Diabetes	21 (23%)
Dyslipidemia	33 (36%)
Atrial fibrillation	45 (49%)
Antiplatelet use	20 (22%)
Anticoagulant use	17 (19%)
Modified Rankin Scale	0 (0–3)
NIHSS score	18 ± 8
Stroke subtype	
Cardioembolic	59 (65)
Large artery atherosclerosis	12 (13)
Other*	20 (22)
Leukocyte count, /μL	7,882 ± 2794
Platelet count, ×10^3^/μL	208 ± 100
PT-INR	0.99 (0.94–1.06)
ASPECTS	7 (4–9)
Occluded vessels	
Extracranial internal carotid artery	4 (4)
Intracranial internal carotid artery	25 (27)
The horizontal segment of MCA	56 (62)
The insular segment of MCA	6 (6)
Intravenous alteplase	28 (31)
Mechanical thrombectomy	54 (59%)
Onset-to-door time, min	155 (72–412)
Onset-to-puncture time, min†	204 (149–311)
Door-to-reperfusion time, min†	143 (108–185)
Puncture-to-reperfusion time, min†	70 (49–98)
Reperfusion within 36h of admission	67 (77%)

Data are presented as median (interquartile range), mean ± standard deviation, or number (percentage). ASPECTS = Alberta Stroke Programme Early CT Score; MCA = middle cerebral artery; NIHSS = National Institutes of Health Stroke Scale.

*Other causes and undetermined etiology.

†Only among patients who underwent mechanical thrombectomy.

### Primary outcome

**Fig 2:**
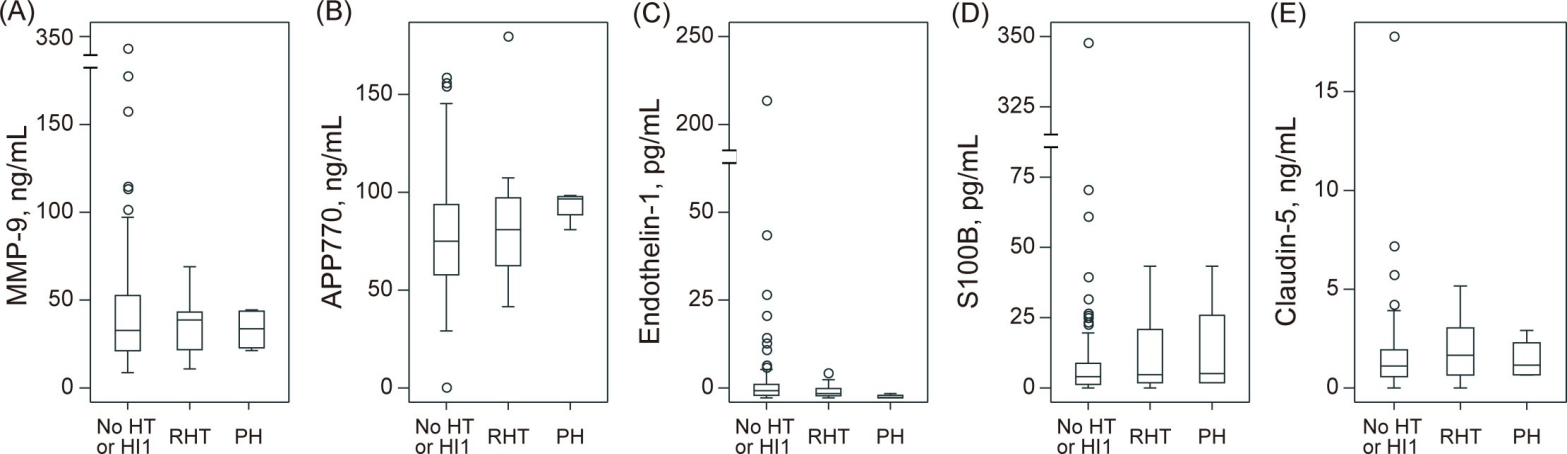
Association between hemorrhagic transformation and biomarkers. There was no significant association between hemorrhagic transformation type and serum biomarker levels. APP, amyloid precursor protein; HI, hemorrhagic infarction; HT, hemorrhagic transformation; MMP-9, matrix metalloproteinase-9; PH, parenchymal hematoma; RHT, relevant hemorrhagic transformation.

Relevant hemorrhagic transformation was observed in 17 (19%) patients, and parenchymal hematoma, 4 (4%) patients. The levels of serum biomarkers according to the hemorrhagic transformation type are shown in Fig 2. MMP-9 (no hemorrhagic transformation or HI1: 46 ± 48 vs. relevant hemorrhagic transformation: 15 ± 4 ng/mL, P = 0.30), APP770 (80 ± 31 vs. 85 ± 8 ng/mL, P = 0.53), endothelin-1 (7.0 ± 25.7 vs. 2.0 ± 2.1 pg/mL, P = 0.42), S100B (13 ± 42 vs. 12 ± 15 pg/mL, P = 0.97), and claudin-5 (1.7 ± 2.3 vs. 1.9 ± 1.5 ng/mL, P = 0.68) levels on admission were not associated with the development of relevant hemorrhagic transformation. Moreover, neither MMP-9 (44 ± 44 vs. 33 ± 12 ng/mL, P = 0.62), APP770 (81 ± 32 vs. 93 ± 8 ng/mL, P = 0.44), endothelin-1 (6.3 ± 23.7 vs. 0.4 ± 0.6 pg/mL, P = 0.62), S100B (13 ± 39 vs. 14 ± 20 pg/mL, P = 0.96), nor claudin-5 (1.7 ± 2.2 vs. 1.5 ± 1.1 ng/mL, P = 0.82) levels were associated with parenchymal hematoma. In patients who underwent mechanical thrombectomy, the level of claudin-5 was higher in patients with relevant hemorrhagic transformation than those without (1.2 ± 1.0 vs. 2.1 ± 1.7 ng/mL, P = 0.0181, S1 Fig).

There was no significant association between the distribution of the techniques used as the first pass and hemorrhagic transformation (P = 0.64). On the contrary, patients who underwent ≥3 passes had more relevant hemorrhagic transformation than those with <3 passes (44% vs. 8%, P = 0.004, S2 Fig).

### Secondary outcomes

**Fig 3:**
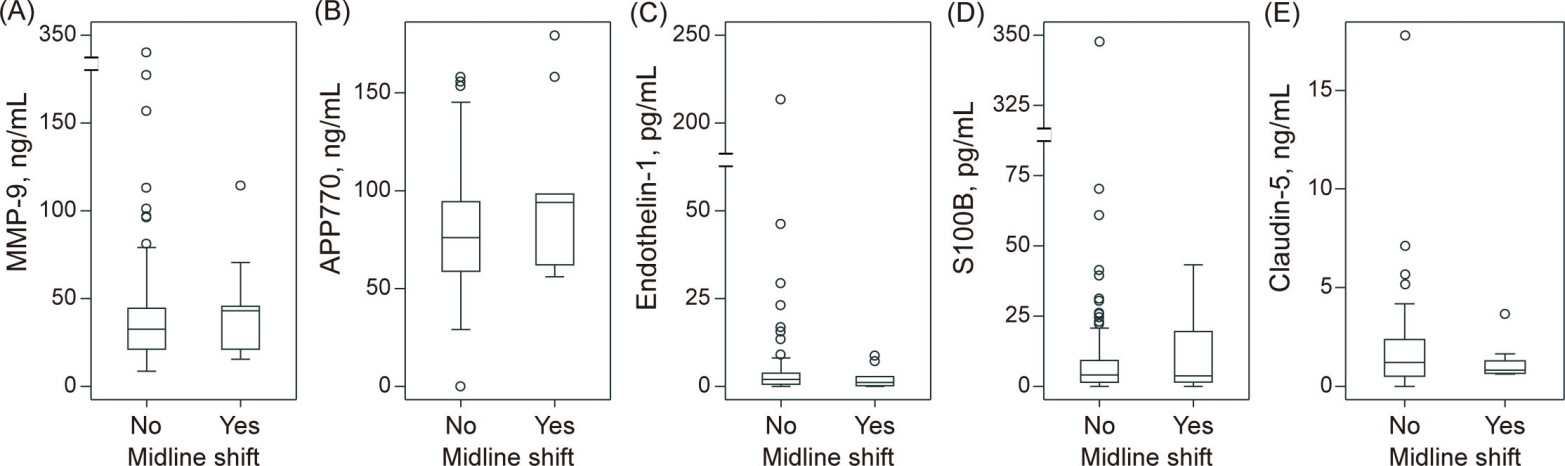
Association between brain edema and biomarkers. APP770 levels were marginally higher in patients with a midline shift ≥ 5 mm than in those without (79 ± 29 vs. 97 ± 41 ng/mL, P = 0.084). APP for amyloid precursor protein; MMP-9, matrix metalloproteinase-9.

Malignant edema, defined as the presence of midline shift ≥ 5 mm, was observed in 10 (11%) patients. The levels of serum biomarkers according to the presence or absence of a midline shift are shown in Fig 3. Neither MMP-9 (without midline shift: 44 ± 45 vs. with midline shift: 46 ± 29 ng/mL, P = 0.92), endothelin-1 (6.5 ± 2.6 vs. 2.4 ± 3.1 pg/mL, P = 0.59), S100B (13 ± 40 vs. 12 ± 16 pg/mL, P = 0.93), nor claudin-5 (1.8 ± 2.3 vs. 1.2 ± 0.9 ng/mL, P = 0.45) levels on admission predicted the development of midline shift, while the APP770 level was marginally higher in patients with midline shift than those without (79 ± 29 vs. 97 ± 41 ng/mL, P = 0.084). Among patients who achieved successful reperfusion, the levels of APP770 were higher in patients with midline shift than in those without (75 ± 26 vs. 118 ± 41 ng/mL, P = 0.003, S3 Fig). The association between baseline characteristics and APP770 levels is shown in S4 Fig. The levels of APP770 were negatively correlated with age (r = -0.32, P = 0.002); however, there was no significant association between the levels of APP770 and NIHSS score (r = -0.05, P = 0.62) or ASPECTS (r = -0.05, P = 0.63).

**Fig 4:**
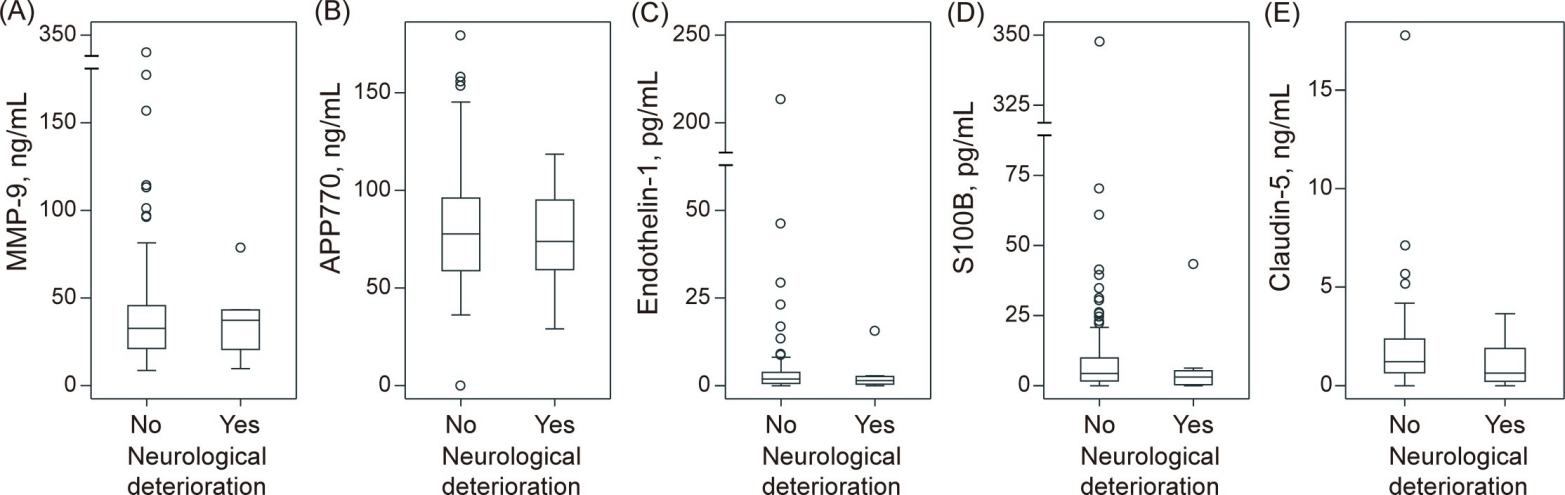
Association between neurological deterioration and biomarkers. There was no significant association between neurological deterioration and the levels of serum biomarkers. APP for amyloid precursor protein; MMP-9, matrix metalloproteinase-9.

Neurological deterioration was observed in 8 (9%) patients. The levels of serum biomarkers according to the presence or absence of neurological deterioration are shown in Fig 4. Neither MMP-9 (45 ± 45 vs. 36 ± 21 ng/mL, P = 0.61), APP770 (82 ± 32 vs. 76 ± 28 ng/mL, P = 0.59), endothelin-1 (6.4 ± 24.2 vs. 3.1 ± 5.2 pg/mL, P = 0.70), S100B (13 ± 40 vs. 7 ± 15 pg/mL, P = 0.69), nor claudin-5 (1.8 ± 2.3 vs. 1.1 ± 1.3 ng/mL, P = 0.45) levels on admission was associated with neurological deterioration.

**Fig 5:**
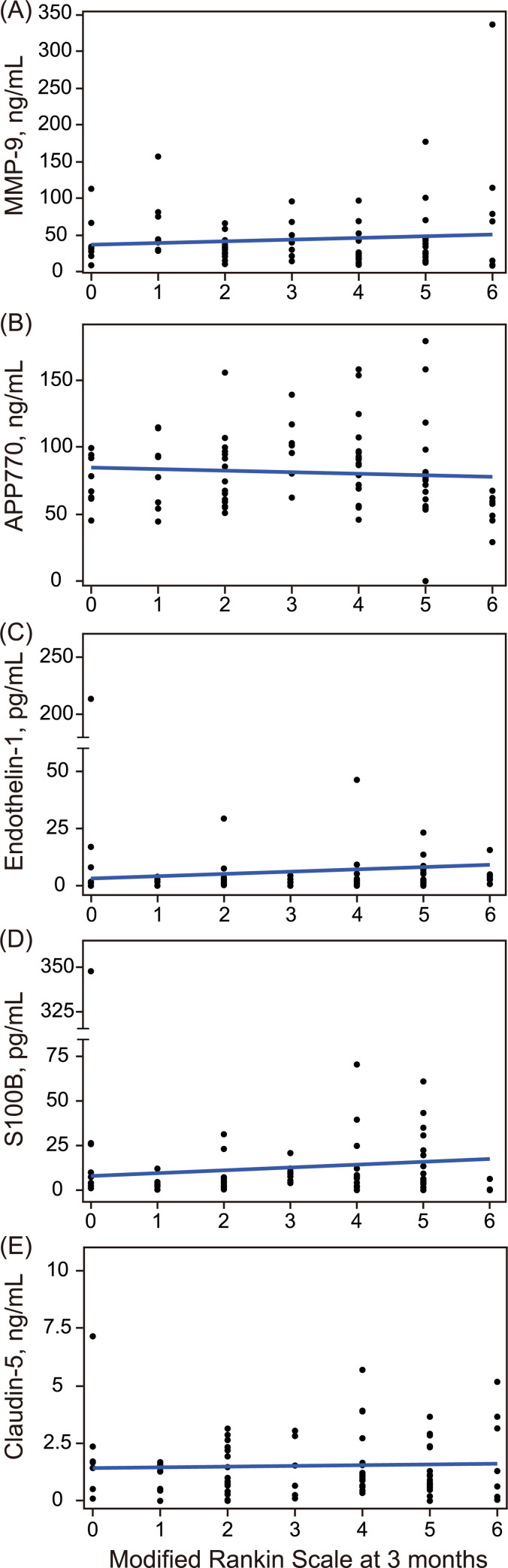
Association between functional outcome and biomarkers. There was no significant association between the modified Rankin Scale score at three months after stroke and serum biomarker levels.

The association between the modified Rankin Scale score and serum biomarkers is shown in Fig 5. Neither MMP-9 (r = 0.09, P = 0.42), APP770 (r = -0.06, P = 0.60), endothelin-1 (r = 0.07, P = 0.51), S100B (r = 0.07, P = 0.52), nor claudin-5 (r = 0.04, P = 0.75) levels on admission were associated with functional outcomes. There was no significant association between serum biomarkers and favorable outcome (S1 Table).

## Discussion

In this single-center prospective study, we examined whether the levels of serum MMP-9, APP770, endothelin-1, S100B, and claudin-5 predict the development of hemorrhagic transformation after large vessel occlusion stroke. Contrary to our expectation, none of these biomarkers were significantly associated with hemorrhagic transformation in our entire cohort, except that the level of claudin-5 was higher in patients with relevant hemorrhagic transformation than in those without when limited to patients who underwent mechanical thrombectomy. The levels of APP770 were marginally associated with severe brain edema. Our study also revealed that none of these biomarkers was associated with functional outcomes.

The usefulness of serum biomarkers for predicting hemorrhagic transformation and edema has been previously reported [11–16]. MMP-9 is a proteolytic enzyme that degrades the endothelial basal lamina and plays a key role in producing edema and hemorrhagic transformation [26–28]. The overexpression of the vasoconstrictor endothelin-1 leads to brain edema, and is a possible biomarker of BBB disruption [15, 29]. S100B is a calcium-binding protein, and it is known that the elevation of serum S100B level reflects BBB damage because the concentration of the protein is much lower than that of cerebrospinal fluid [30, 31]. Claudin-5 is a tight junction protein, and is one of the structural components of the BBB [11]. It seems reasonable that these biomarkers are associated with the course after cerebral infarction. However, except for Claudin-5, we failed to demonstrate the predictive role of these serum biomarkers for hemorrhagic transformation in the era of mechanical thrombectomy. There are several reasons for this. Among patients who underwent mechanical thrombectomy, the degree of direct vessel wall damage by endovascular procedures may be more important than BBB damage due to ischemia in this context; more than three passes of stent retriever predict parenchymal hematoma [32], and the rate of symptomatic intracranial hemorrhage or parenchymal hematoma exponentially increases with the elapsed time after puncture [33]. Also, the relatively low number of patients who received intravenous rt-PA may have affected the results, because rt-PA is known to activate MMP-9 [34].

In the present study, the levels of APP770 were marginally higher among patients with a midline shift than in those without. APP770 is a different APP isoform than the neuronal APP695. APP770 is secreted by inflamed endothelial cells and activated platelets [35]. Among patients with small subcortical infarcts, we have previously reported that APP770 levels are higher in patients with progressive neurological deficits than in those without, suggesting that APP770 might be a biomarker of cerebral small vessel disease [36]. We measured APP770 levels in patients with large vessel occlusion with the assumption that they reflect endothelial dysfunction. Our findings suggest that the levels of APP770 on admission may predict the extent of reperfusion injury, as the association between APP770 and brain edema was stronger in patients who achieved successful reperfusion than in those who did not.

Space-occupying cerebral edema subsequent to a large infarction can lead to neurologic deterioration and brain herniation. The natural course in patients with malignant edema is disastrous, with mortality rates up to 80% [37]. Reperfusion, especially when late, may augment brain edema [4, 5]. Therefore, predicting brain edema may help to decide who should not undergo reperfusion therapy. In addition, as early decompressive surgery has been shown to reduce mortality and disability in patients with malignant edema, it is important to identify patients who are at risk for developing malignant edema. Further studies are needed to determine the predictive value of serum APP770 levels for brain edema in patients with large vessel occlusion stroke.

Some serum biomarkers have been reported in patients who do not undergo mechanical thrombectomy, but they were not evaluated in the present study. Cellular fibronectin and platelet-derived growth factor-CC are known predictors of hemorrhagic transformation [13, 38]. However, we were unable to measure them appropriately. A high serum homocysteine level and low Caveolin-1 level have been reported as independent predictors of hemorrhagic transformation [39]. Furthermore, some non-serologic biomarkers have been reported in patients who undergo mechanical thrombectomy. For example, a high admission neutrophil-to-lymphocyte ratio has been reported as an independent predictor of symptomatic intracranial hemorrhage after mechanical thrombectomy [40, 41]. In addition, patient age, smoking, ASPECTS, general anesthesia, and embolization in a new territory are reportedly associated with hemorrhagic transformation [9]. Poor collateral status is also associated with hemorrhagic transformation [9, 42]. Using serum biomarkers in combination with these biomarkers may contribute to accurate prediction of patients who are at a higher risk of hemorrhagic transformation.

This study has several limitations. First, the number of patients from whom informed consent was obtained was lower than to those who were screened. This is due to the difficulty in obtaining written informed consent in hyperacute situations. Selection bias may have affected our findings. Second, we did not evaluate the correlation between serum biomarkers and perfusion imaging, although perfusion status is reported to be associated with hemorrhagic transformation [43]. Finally, this study may be underpowered, and multivariate analysis could not be performed due to the limited sample size.

## Conclusions

Previously reported serum biomarkers did not predict hemorrhagic transformation in this cohort of patients with large vessel occlusion stroke, except that the level of claudin-5 was higher in patients with relevant hemorrhagic transformation than in those without in the subpopulation who underwent mechanical thrombectomy. On the other hand, the levels of APP770 were marginally associated with brain edema. Further studies are needed to identify the best biomarker for hemorrhagic transformation in the era of mechanical thrombectomy.

## Supporting information

S1 Fig Association between hemorrhagic transformation and biomarkers only in patients who underwent mechanical thrombectomy. The level of claudin-5 was higher in patients with relevant hemorrhagic transformation than those without (1.2 ± 1.0 vs. 2.1 ± 1.7 ng/mL; P = 0.0181). APP, amyloid precursor protein; HI, hemorrhagic infarction; HT, hemorrhagic transformation; MMP-9, matrix metalloproteinase-9; PH, parenchymal hematoma; RHT, relevant hemorrhagic transformation.
(DOCX)

S2 Fig Association between hemorrhagic transformation and number of device passes. Patients who underwent ≥3 passes had more relevant hemorrhagic transformation than those with <3 passes (44% vs. 8%, P = 0.004). HT, hemorrhagic transformation.
(DOCX)

S3 Fig Association between brain edema and serum APP770 according to the presence or absence of successful reperfusion. Among patients who achieved successful reperfusion, the levels of APP770 were higher in patients with midline shift than in those without (75 ± 26 vs. 118 ± 41 ng/mL, P = 0.003).
(DOCX)

S4 Fig Association between the level of APP770 and patients’ characteristics. The levels of APP770 were negatively correlated with age (r = -0.32, P = 0.002). Among patients who achieved successful reperfusion at 24 ± 12 hours after admission, the levels of APP770 were higher in patients with a midline shift ≥ 5 mm than in those without (75 ± 26 vs. 118 ± 41 ng/mL, P = 0.003).
(DOCX)

S1 Table Association between favorable outcome and biomarkers. (DOCX)
