# Supplementary material for: Predicting hemorrhagic transformation after large vessel occlusion stroke in the era of mechanical thrombectomy
Source: PLoS One. 2021 Aug 16;16(8):e0256170. doi: 10.1371/journal.pone.0256170 (PMC8366990; doi:10.1371/journal.pone.0256170)
Supplement: S1 Fig — The level of claudin-5 was higher in patients with relevant hemorrhagic transformation than those without (1.2 ± 1.0 vs. 2.1 ± 1.7 ng/mL; P = 0.0181). APP, amyloid precursor protein; HI, hemorrhagic infarction; HT, hemorrhagic transformation; MMP-9, matrix metalloproteinase-9; PH, parenchymal hematoma; RHT, relevant hemorrhagic transformation. (DOCX) [file pone.0256170.s001.docx]

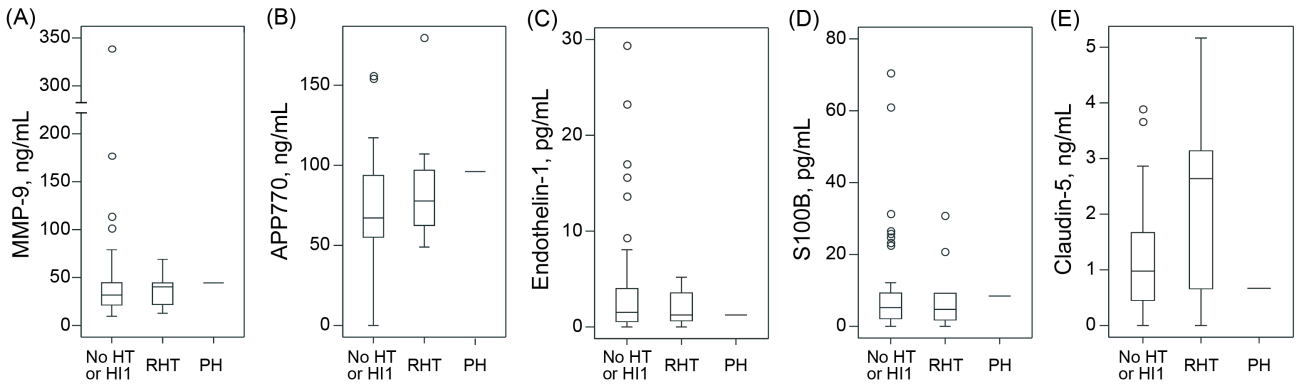


**S1 Fig. Association between hemorrhagic transformation and biomarkers only in patients who underwent mechanical thrombectomy**

The level of claudin-5 was higher in patients with relevant hemorrhagic transformation than those without (1.2 ± 1.0 vs. 2.1 ± 1.7 ng/mL; P = 0.0181). APP, amyloid precursor protein; HI, hemorrhagic infarction; HT, hemorrhagic transformation; MMP-9, matrix metalloproteinase-9; PH, parenchymal hematoma; RHT, relevant hemorrhagic transformation.
