# Supplementary material for: Predicting hemorrhagic transformation after large vessel occlusion stroke in the era of mechanical thrombectomy
Source: PLoS One. 2021 Aug 16;16(8):e0256170. doi: 10.1371/journal.pone.0256170 (PMC8366990; doi:10.1371/journal.pone.0256170)
Supplement: S2 Fig — Patients who underwent ≥3 passes had more relevant hemorrhagic transformation than those with <3 passes (44% vs. 8%, P = 0.004). HT, hemorrhagic transformation. (DOCX) [file pone.0256170.s002.docx]

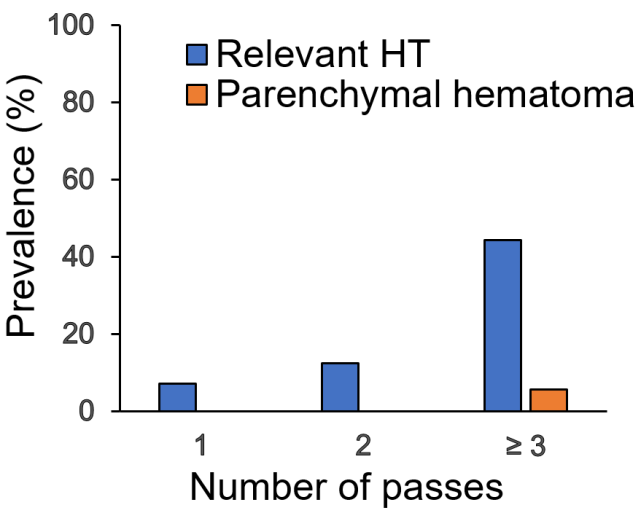


**S2 Fig. Association between hemorrhagic transformation and number of device passes**

Patients who underwent ≥3 passes had more relevant hemorrhagic transformation than those with <3 passes (44% vs. 8%, P = 0.004).

HT, hemorrhagic transformation.
