# Supplementary material for: Predicting hemorrhagic transformation after large vessel occlusion stroke in the era of mechanical thrombectomy
Source: PLoS One. 2021 Aug 16;16(8):e0256170. doi: 10.1371/journal.pone.0256170 (PMC8366990; doi:10.1371/journal.pone.0256170)
Supplement: S3 Fig — Among patients who achieved successful reperfusion, the levels of APP770 were higher in patients with midline shift than in those without (75 ± 26 vs. 118 ± 41 ng/mL, P = 0.003). (DOCX) [file pone.0256170.s003.docx]

**
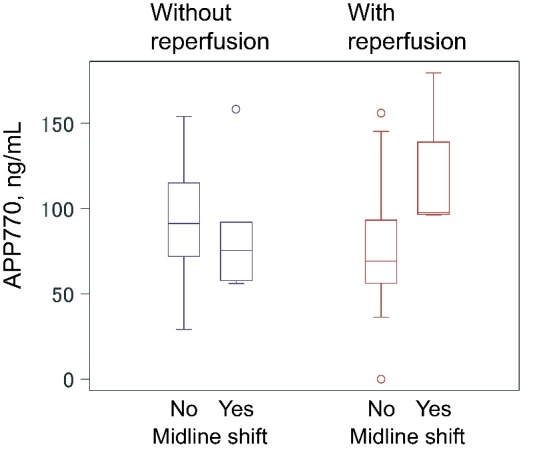
**

**S3 Fig. Association between brain edema and serum APP770 according to the presence or absence of successful reperfusion**

Among patients who achieved successful reperfusion, the levels of APP770 were higher in patients with midline shift than in those without (75 ± 26 vs. 118 ± 41 ng/mL, P = 0.003).
