# Supplementary material for: Predicting hemorrhagic transformation after large vessel occlusion stroke in the era of mechanical thrombectomy
Source: PLoS One. 2021 Aug 16;16(8):e0256170. doi: 10.1371/journal.pone.0256170 (PMC8366990; doi:10.1371/journal.pone.0256170)
Supplement: S4 Fig — The levels of APP770 were negatively correlated with age (r = -0.32, P = 0.002). Among patients who achieved successful reperfusion at 24 ± 12 hours after admission, the levels of APP770 were higher in patients with a midline shift ≥ 5 mm than in those without (75 ± 26 vs. 118 ± 41 ng/mL, P = 0.003). (DOCX) [file pone.0256170.s004.docx]

**
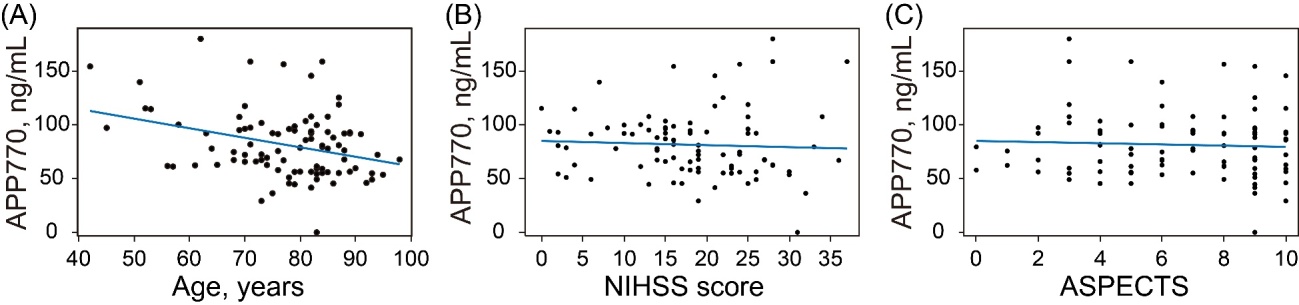
**

**S4 Fig. Association between the level of APP770 and patients characteristics**

The levels of APP770 were negatively correlated with age (r = -0.32, P = 0.002). Among patients who achieved successful reperfusion at 24 ± 12 hours after admission, the levels of APP770 were higher in patients with a midline shift ≥ 5 mm than in those without (75 ± 26 vs. 118 ± 41 ng/mL, P = 0.003).
