# Supplementary material for: Predicting hemorrhagic transformation after large vessel occlusion stroke in the era of mechanical thrombectomy
Source: PLoS One. 2021 Aug 16;16(8):e0256170. doi: 10.1371/journal.pone.0256170 (PMC8366990; doi:10.1371/journal.pone.0256170)
Supplement: S1 Table — (DOCX) [file pone.0256170.s005.docx]

| **S1 Table. Association between favorable outcome and biomarkers.** | | | |
| --- | --- | --- | --- |
| Biomarker | Odds Ratio | 95% CI | P |
| MMP-9, per 10 ng/mL increase | 0.98 | 0.89–1.09 | 0.71 |
| APP770, per 10 ng/mL increase | 0.96 | 0.83–1.11 | 0.57 |
| Endothelin-1, per 10 pg/mL increase | 0.73 | 0.34–1.54 | 0.183 |
| S100B, per 10 pg/mL increase | 0.74 | 0.48–1.13 | 0.162 |
| Claudin-5, per 1 ng/mL increase | 0.91 | 0.66–1.27 | 0.59 |
| APP, amyloid precursor protein; CI, confidence interval; MMP-9, matrix metalloproteinase-9. | | | |
